# Daily rainfall nearest neighbor pattern using point data series in Iran

**DOI:** 10.1016/j.dib.2018.06.021

**Published:** 2018-06-19

**Authors:** Majid Javari

**Affiliations:** College of Social Science, PayameNoor University PO BOX 19395-3697, Tehran, Iran

**Keywords:** Point data, Daily rainfall, Variability patterns and nearest neighbor׳s patterns

## Abstract

In this data study, assessment of daily rainfall nearest neighbor׳s patterns (DRBBP) was described in Iran. This article presents some spatial patterns of daily rainfall nearest neighbor׳s patterns for Iran from 170 stations and 31195 rainfall points by comparing ordinary kriging techniques based on the forecast models. For the nearest neighbor׳s patterns of the daily rainfall, rainfall data series of 1975–2014 was employed to estimate the point data of daily rainfall. The statistical properties were analyzed to indicate an increase in dispersed variability patterns of daily rainfall in Iran. Dispersed patterns were selected as the best nearest neighbor׳s models to model daily rainfall variability. The data results will help climatologists and hydrologists in model assessment and planning of natural environment in Iran.

**Specifications Table**

**Table:** 

Subject area	Climatology and Atmospheric environment
More specific subject area	Spatial statistics, GIS applications, Environmental science
Type of data	Table, figure, point and raster
How data was acquired	GIS_ based extracted data
Data format	Raster, matrix, extracted, and analyzed
Experimental factors	Data collected from Meteorological Organization of Iran (IRIMO) and GIS_ based extracted data
Experimental features	GIS_ based and spatial statistical Analysis
Data source location	Data collected from Meteorological Organization of Iran (IRIMO) and GIS_ based extracted data
Data availability	All data included in this article

**Value of the data**

• The data could be suitable for climatic analysis in predicting of rainfall variability for climate classification in Iran.
• The data base could provide viewpoints of variability patterns of the daily rainfall using nearest neighbor׳s techniques in Iran.
• The dataset will help climatologists and hydrologists in model fitting and planning of natural environment in Iran.
• The data will be helpful in predicting of the variability patterns of the daily rainfall through which the environmental planning.

## 1 Data

The rainfall data series for this study was extracted from the Meteorological Organization of Iran (IRIMO) from 1975 through 2014 from 170 synoptic and climatology stations in Iran. The point dataset to estimate the daily rainfall nearest neighbor׳s patterns (DRBBP) for all stations is extracted from a rainfall dataset of the stations. Rainfall data series obtained from the IRIMO differs from rainfall datasets predicted by the ordinary kriging in the data distributions are estimated on models of the ordinary kriging. In addition, rainfall validation datasets (RVD) were extracted from ordinary kriging models based on its spatial coordinates calculated by [1], [2]:

$$Y_{\left(S\right)}=\beta_{0}+\beta_{1}x+\beta_{2}y+\beta_{3}x^{2}+\beta_{4}y^{2}+\beta_{5}\mathrm{xy}$$

where $Y_{\left(S\right)}$ this is a second-order polynomial trend surface on the spatial x- and y-coordinates. The point dataset collected are used based on observed mean distance, expected mean distance, nearest neighbor index, z-score, and p-value to assess the nearest neighbor׳s patterns. However, the point daily rainfall dataset across the rainfall points 31195 is a suitable tool to analyze the nearest neighbor׳s patterns. DRBBP depends on dataset extracted methods such as the ordinary kriging, and the point average nearest neighbor (PANN) is defined as [3]:

$$\mathrm{PANN}=\frac{\mathrm{OMD}}{\mathrm{EMD}}=\frac{\sum_{i=1}^{n}d_{i}/n}{0.5/\sqrt{n/A}}$$

OMD is the observed mean distance between each rainfall point and its nearest neighbor, EMD is the expected mean distance in a random distribution, $d_{i}$ is the distance between rainfall point and its nearest neighboring, n is the total number of rainfall points, and A is the area of a minimum enclosing rectangle around all rainfall points. For the average nearest neighbor ratio (Moran׳s I index), a Moran׳s I index value less than 1 shows a pattern clustering (less spatial variability), while a Moran׳s I index value greater than 1 indicates a dispersion pattern (more spatial variability). In this analysis, Moran׳s I index value equal to 1, shows a random pattern the statistically significant the random of daily rainfall series. The quality and characteristics of the point dataset can be studied using spatial statistical methods tools such as calculating areas(for each rainfall point in a polygon rainfall points dataset), estimating distance band from neighbor count (the minimum, maximum, and average distance to the specific Nth nearest neighbor), switching rainfall points data (transfers rainfall points to weighted rainfall point data), and converting spatial weights matrix to table (transforms a binary spatial weights matrix file to a table) for a dataset of rainfall points. The rainfall point dataset is a climatic time series affecting the climatic variability in the climate classification. Rainfall point data sets were extracted from the GIS-based layers using the ordinary kriging based on the Circular, Spherical, Tetraspherical, Pentaspherical, Exponential, Gaussian, Rational Quadratic, Hole Effect, K-Bessel, J-Bessel, and Stable models. Therefore, the estimated daily rainfall dataset over the forty years is a suitable typical data series of the spatial variability of RVD as revealed in Tables 1–5. The nature and characteristics of the daily rainfall dataset can be studied using spatial statistical methods tools such as daily rainfall nearest neighbor׳s patterns (DRBBP).

**Table 1:** Daily rainfall data mean from Ordinary kriging models.

Method	Circular	Spherical	Tetraspherical	Pentaspherical	Exponential	Gaussian	Rational Quadratic	Hole Effect	K-Bessel	J-Bessel	Stable
Mean	0.906	0.9048	0.9052	0.9054	0.9058	0.9016	0.9038	0.9097	0.9035	0.9038	0.9049

**Table 2:** Point daily rainfall data mean from Ordinary kriging models.

Method	Circular	Spherical	Tetraspherical	Pentaspherical	Exponential	Gaussian	Rational Quadratic	Hole Effect	K-Bessel	J-Bessel	Stable
Mean	0.665	0.666	0.667	0.667	0.671	0.665	0.6696	0.667	0.668	0.668	0.669

**Table 3:** The statistical properties of the data extracted from ordinary kriging models.

**Model**	**N**	**Mean**	**SE of mean**	**Interquartile range**	**Standard deviation**	**Variance**	**Coefficient Variations**	**Minimum**	**Maximum**	**Median**	**Skewness**	**Kurtosis**
Circular	170	0.9056	0.0414	0.6525	0.5353	0.2866	59.11	0.2148	3.1019	0.8111	1.31	2.34
Exponential	170	0.9058	0.0401	0.6742	0.5185	0.2688	57.24	0.2141	3.1843	0.8305	1.42	3.24
Gaussian	170	0.9016	0.0426	0.6774	0.5511	0.3037	61.12	0.2125	3.2318	0.7866	1.41	2.62
Hole Effect	170	0.9098	0.0387	0.6683	0.5003	0.2503	54.99	0.2128	2.6599	0.8587	0.96	0.95
J-Bessel	170	0.9039	0.042	0.6724	0.5431	0.295	60.09	0.2151	3.1515	0.8005	1.39	2.51
K-Bessel	170	0.9035	0.0413	0.6391	0.5331	0.2842	59	0.2123	3.2021	0.8051	1.42	2.94
Pentaspherical	170	0.9058	0.0412	0.6409	0.532	0.283	58.73	0.2141	3.165	0.8199	1.37	2.76
Rational Quadratic	170	0.9038	0.0413	0.6378	0.5338	0.2849	59.06	0.1963	3.2821	0.827	1.51	3.45
Spherical	170	0.9048	0.0412	0.6446	0.5327	0.2838	58.88	0.2155	3.1357	0.8114	1.34	2.55
Stable	170	0.9049	0.0409	0.6437	0.5284	0.2793	58.4	0.2118	3.221	0.8246	1.43	3.16
Tetraspherical	170	0.9052	0.0412	0.6414	0.5326	0.2837	58.84	0.2154	3.1594	0.8202	1.36	2.68

**Table 4:** The statistical properties of the data extracted from point models of the ordinary kriging.

**Model**	**N**	**Mean**	**SE of mean**	**Interquartile range**	**Standard deviation**	**Variance**	**Coefficient Variations**	**Minimum**	**Maximum**	**Median**	**Skewness**	**Kurtosis**
Point_Circular	31195	0.66504	0.00251	0.53112	0.44382	0.19698	66.74	0.17738	3.73058	0.52028	1.79	4.63
Point Exponential	31195	0.6711	0.00247	0.54293	0.43635	0.1904	65.02	0.17291	4.01968	0.51859	1.89	5.43
Point Gaussian	31195	0.665	0.0026	0.5232	0.45863	0.21034	68.97	0.16669	3.89062	0.50801	1.95	5.51
Point Hole Effect	31195	0.66737	0.00244	0.5662	0.43065	0.18546	64.53	0.15987	3.22307	0.52491	1.49	2.94
Point J-Bessel	31195	0.66815	0.00257	0.51501	0.45395	0.20607	67.94	0.13191	3.86073	0.51208	1.94	5.42
Point K-Bessel	31195	0.66819	0.00253	0.53295	0.44678	0.19961	66.86	0.17617	3.78929	0.51579	1.91	5.33
Point Pentaspherical	31195	0.66715	0.00252	0.5366	0.44424	0.19735	66.59	0.17822	3.85764	0.51827	1.87	5.14
Point Rational Quadratic	31195	0.66964	0.00253	0.52503	0.44754	0.20029	66.83	0.17525	3.87946	0.51322	1.99	5.85
Point Spherical	31195	0.66585	0.00251	0.53565	0.44409	0.19722	66.69	0.17608	3.78894	0.51935	1.82	4.87
Point Stable	31195	0.66937	0.00251	0.53713	0.4425	0.19581	66.11	0.17481	3.9058	0.51768	1.9	5.41
Point Tetraspherical	31195	0.66665	0.00251	0.53741	0.44419	0.19731	66.63	0.17631	3.82861	0.51882	1.85	5.02

**Table 5:** DRBBP based on the Ordinary kriging models.

Method	Circular	Spherical	Tetraspherical	Pentaspherical	Exponential	Gaussian	Rational Quadratic	Hole Effect	K-Bessel	J-Bessel	Stable
Mean	1.41633	1.41633	1.41633	1.82566	1.20635	1.20635	1.82566	1.82566	1.82566	1.82566	1.82566

### 1.1 The mean estimation of the data from ordinary kriging

**Fig. 1:**
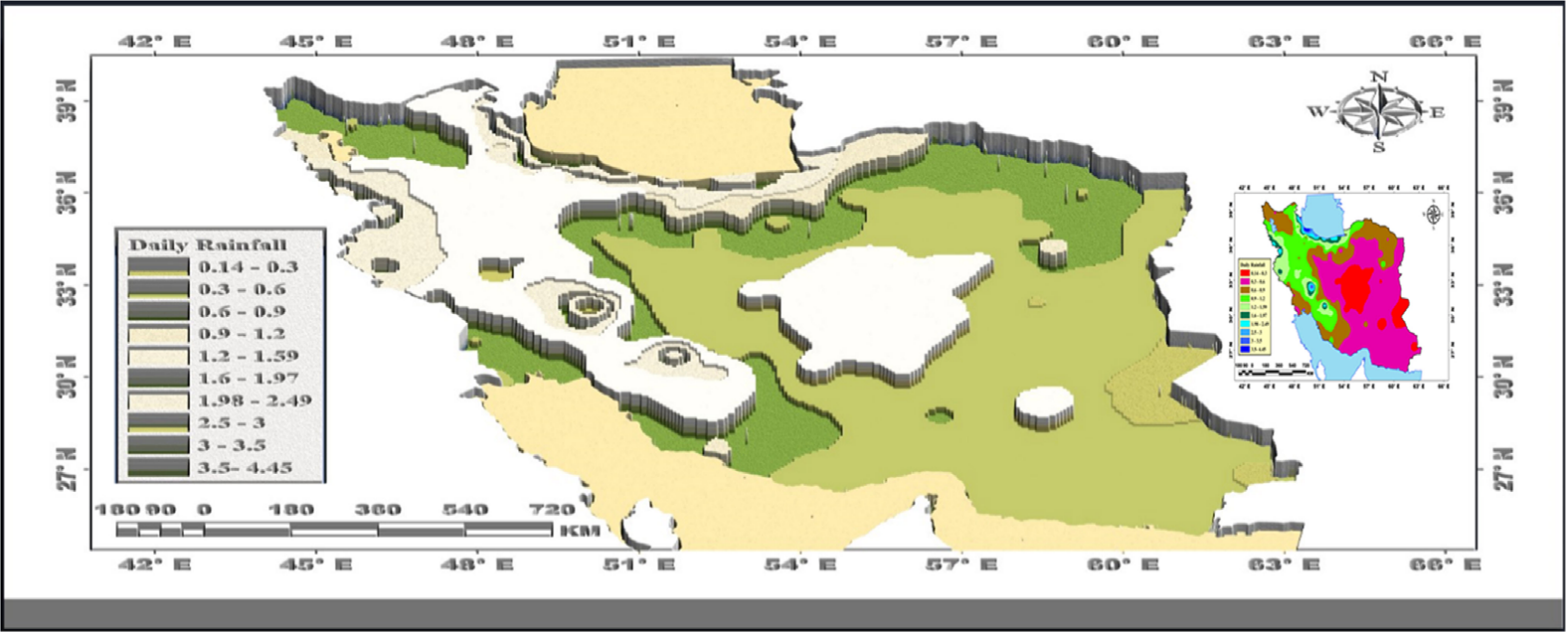
Spatial variability of the daily rainfall using spherical model in Iran.

Table 1 presents the mean estimation of the data extracted from ordinary kriging. Fig. 1 also presents the data. Fig. 1 describes the statistical variability properties revealing the spatial variability of the daily rainfall in Iran.

### 1.2 The mean estimation of the point data from ordinary kriging

**Fig. 2:**
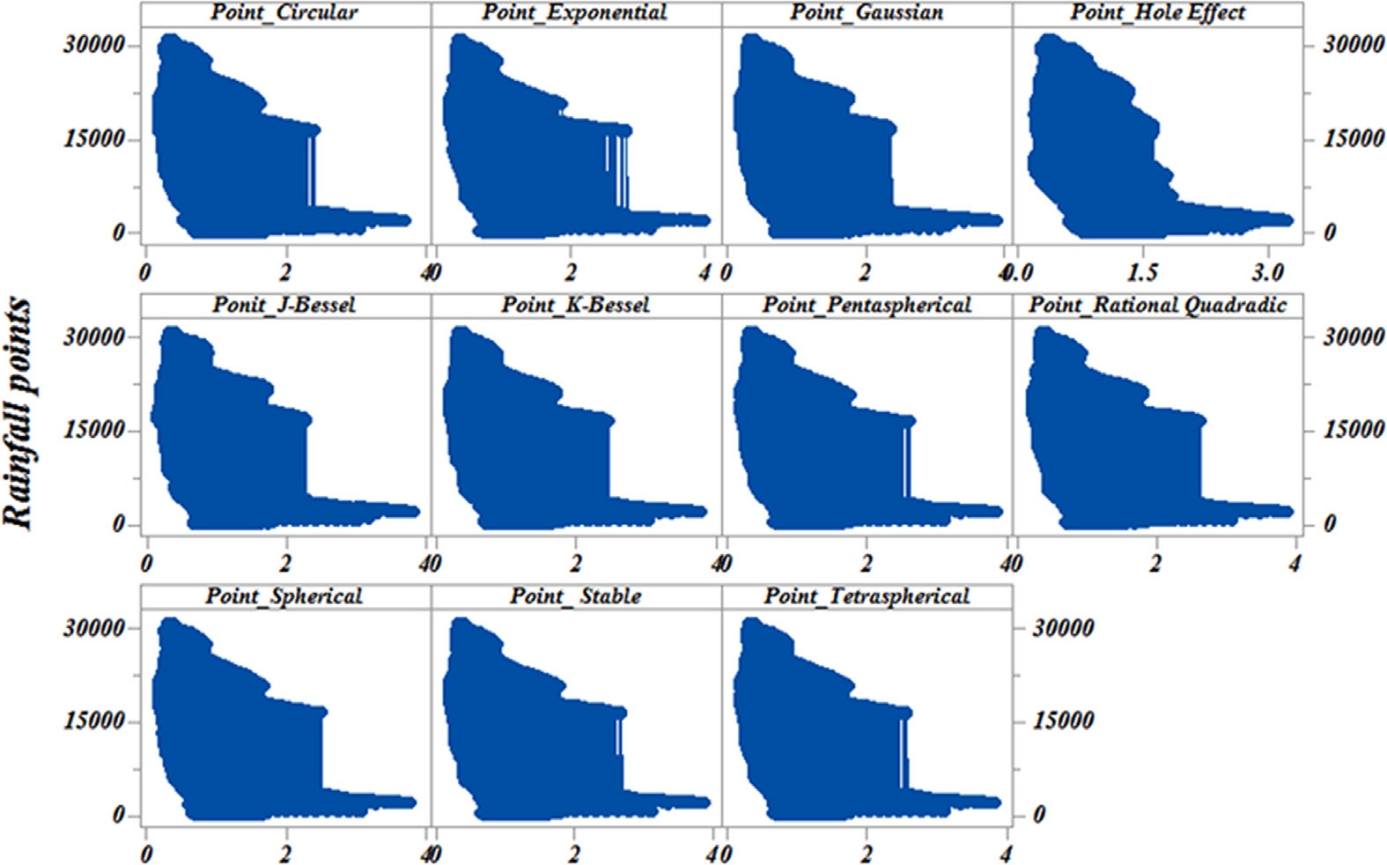
Variability of the point daily rainfall using ordinary kriging models in Iran.

Table 2 presents the mean estimation of the data extracted from ordinary kriging. Fig. 2 also presents the data. Fig. 2 depicts the statistical variability properties revealing the point daily rainfall in Iran.

### 1.3 The statistical properties of the data from ordinary kriging

**Fig. 3:**
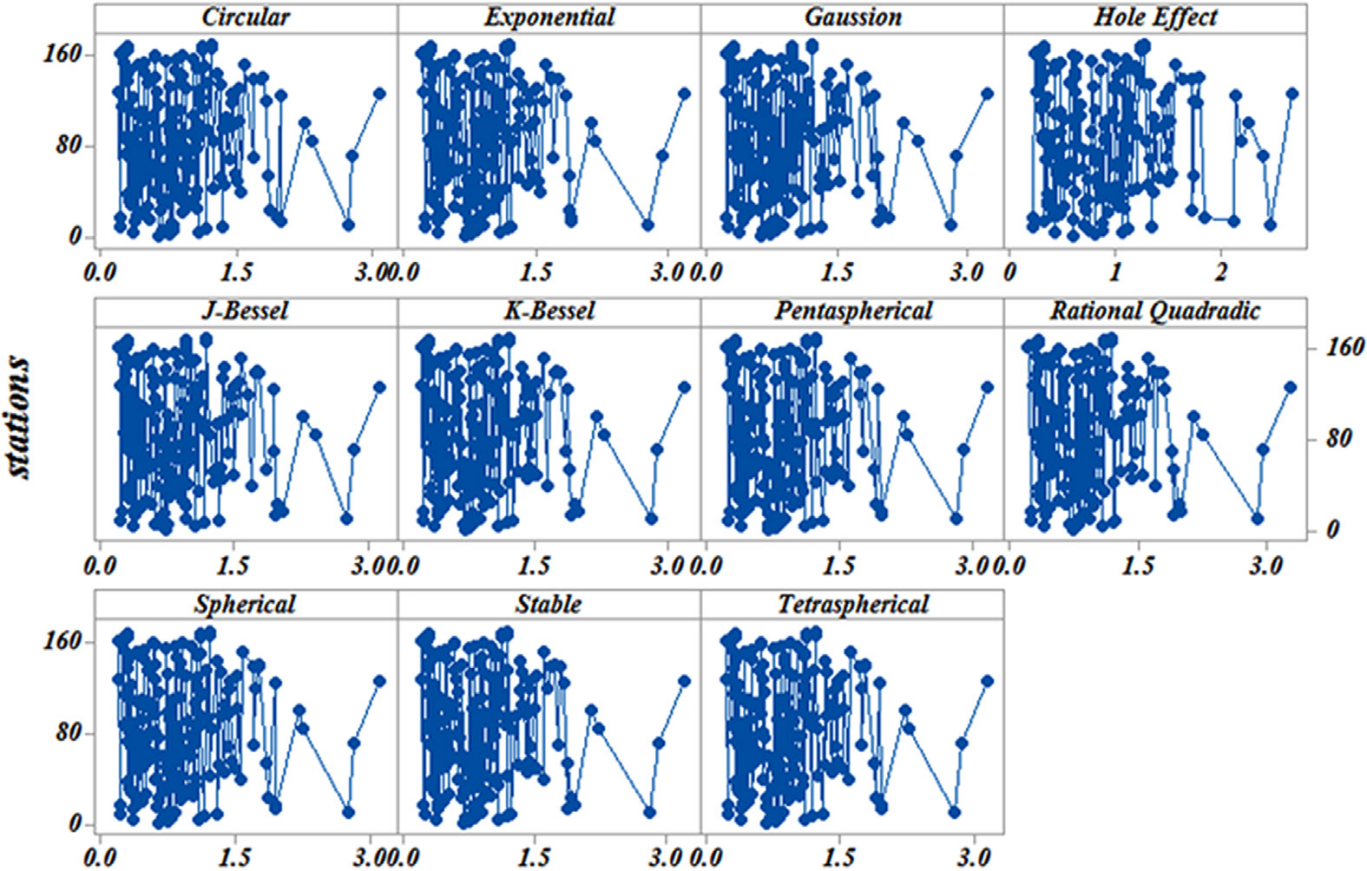
Variability of the daily rainfall using ordinary kriging models in Iran.

Table 3 presents the statistical properties of the data extracted from ordinary. Fig. 3 also presents the data. Fig. 3 describes the statistical variability properties revealing the spatial variability of the daily rainfall in Iran.

### 1.4 The statistical properties of the data from point model of the ordinary kriging

**Fig. 4:**
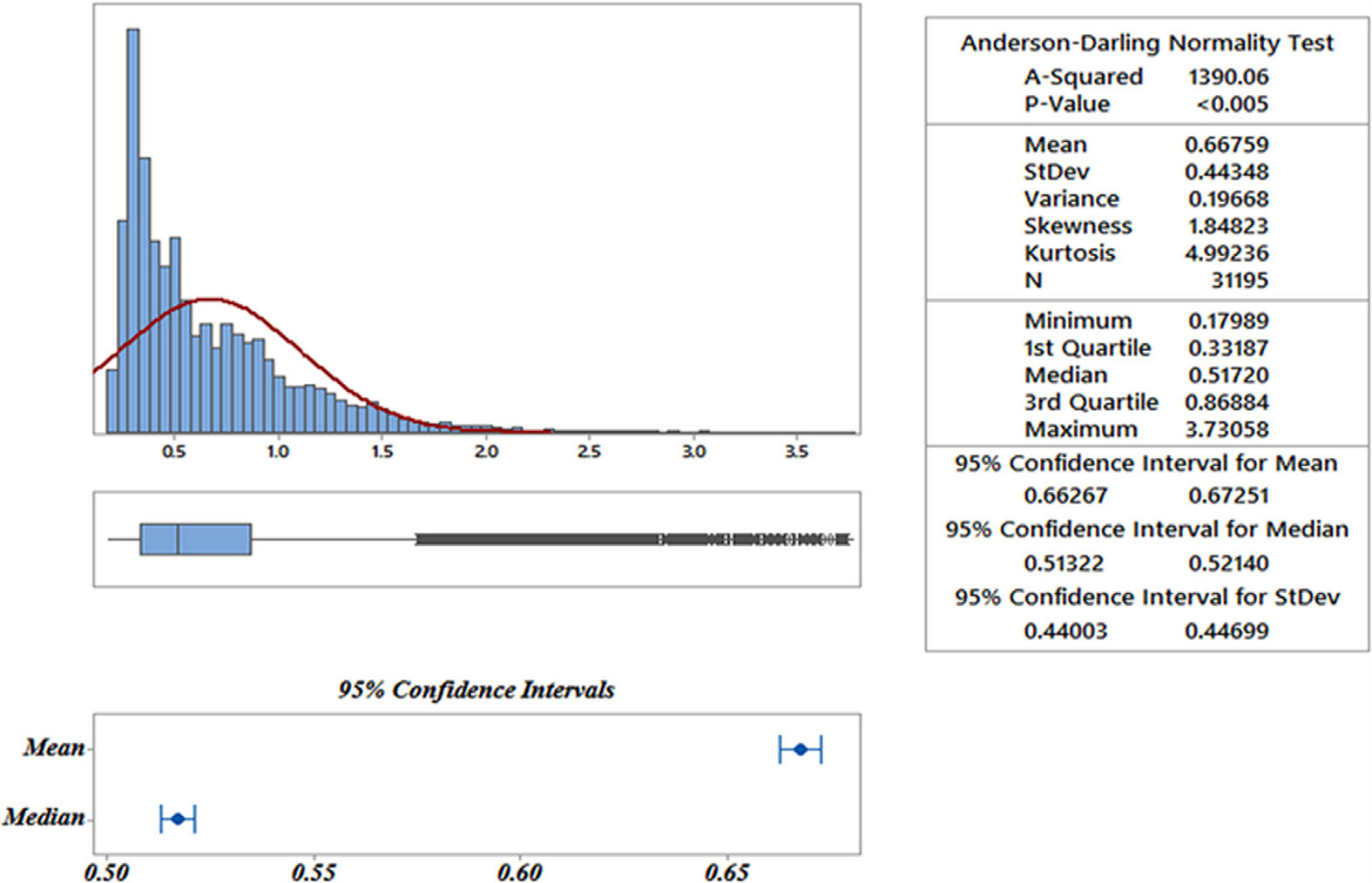
Variability of the point daily rainfall distribution using ordinary kriging models in Iran.

Table 4 presents the statistical properties of the data extracted from point ordinary kriging. Fig. 4 also presents the data. Fig. 4 depicts the statistical distribution variability properties revealing the spatial variability of the abnormal distribution of the daily rainfall in Iran.

### 1.5 The statistical properties of the data from DRBBP

**Fig. 5:**
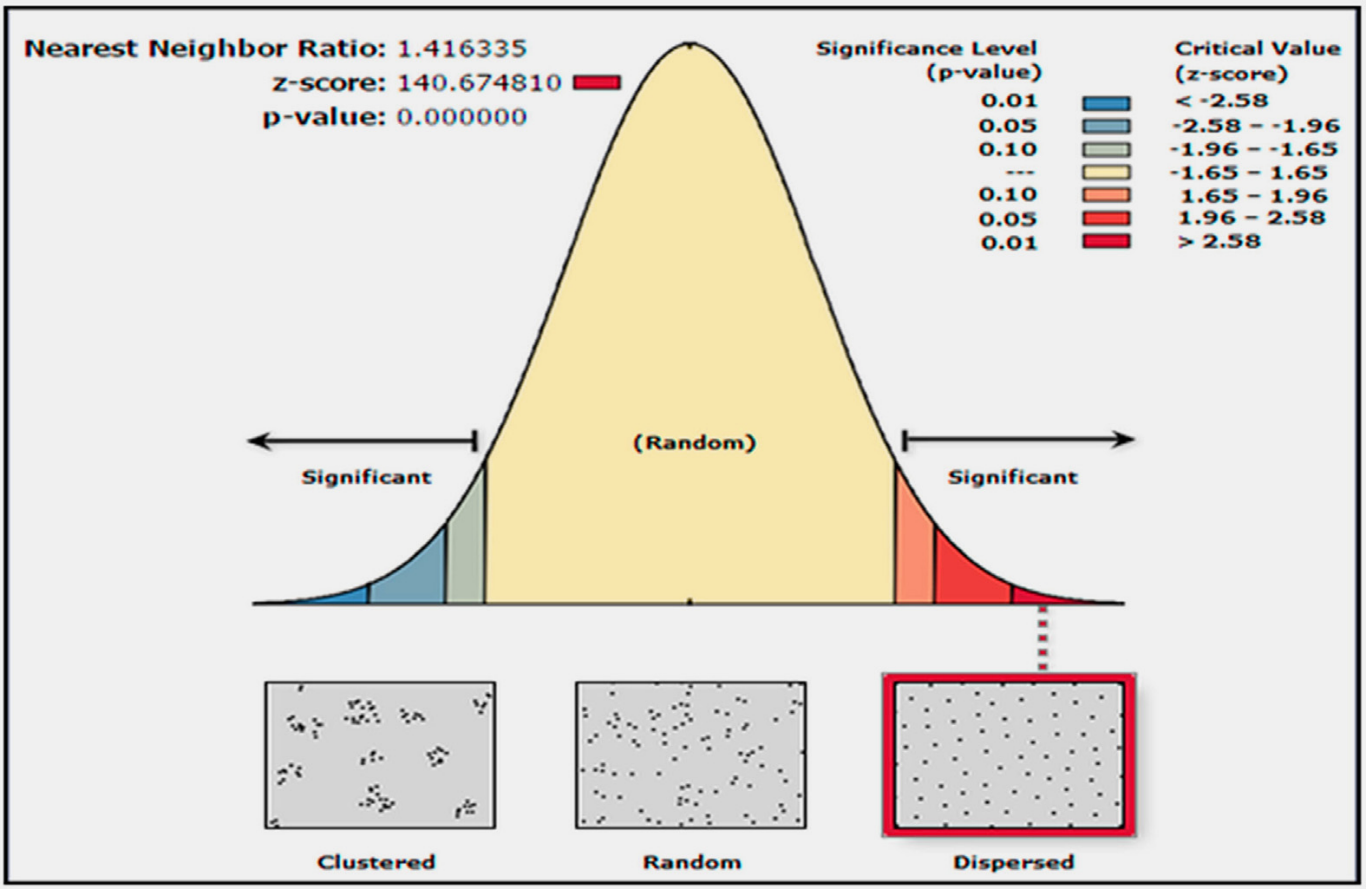
Daily rainfall nearest neighbor׳s index using Point spherical model.

Table 5 presents the statistical estimation of the data extracted from DRBBP in Iran. Fig. 5 also presents the data. Fig. 5 depicts the nearest neighbor׳s patterns properties revealing the dispersed variability pattern of the daily rainfall in Iran.

## 2 Materials and methods

Several studies have been presented on nearest neighbor׳s patterns properties of rainfall [4], [5], [6], [7], [8], [9], [10]. The rainfall data for this analysis were collected from the IRIMO from 1975 through 2014 from 170 synoptic and climatology stations in Iran. The rainfall dataset extracted and compared in the climatology primary data such as daily rainfall point series was employed as recorded variables for the present article result.
