# Supplementary material for: Daily rainfall nearest neighbor pattern using point data series in Iran
Source: Data Brief. 2018 Jun 19;19:1432–40. doi: 10.1016/j.dib.2018.06.021 (PMC6141148; doi:10.1016/j.dib.2018.06.021)
Supplement: Supplementary file 1 — Supplementary material [file mmc1.doc]

***Conflicts of Interest Statement***

**Manuscript title: Daily rainfall nearest neighbor pattern using point data series in Iran**

The author whose name is listed immediately below certify that they have NO affiliations with or involvement in any organization or entity with any financial interest, or non-financial interest in the subject matter or materials discussed in this manuscript.

**Author names: Majid Javari Author's signature** **Date: 2018.06.03**
